# Nurturing nature

**DOI:** 10.7554/eLife.61323

**Published:** 2020-09-09

**Authors:** Elena Dreosti

**Affiliations:** Wolfson Institute for Biomedical Research, University College LondonLondonUnited Kingdom

**Keywords:** social genetic effects, indirect genetic effects, GxE interaction, oxytocin, social behavior, Zebrafish

## Abstract

Mutant zebrafish exhibit different behaviours depending on the genetic background of the fish they were raised with.

**Related research article** Ribeiro D, Nunes AR, Teles M, Anbalagan S, Blechman J, Levkowitz G, Oliveira RF. 2020. Genetic variation in the social environment affects behavioral phenotypes of oxytocin receptor mutants in zebrafish. *eLife* **9**:e56973. doi: 10.7554/eLife.56973

Behaviours emerge under the combined influence of the environment (nurture) and the genetic information an individual inherited from its ancestors (nature). However, it is still difficult to tease apart the respective contribution of these different factors, which are often deeply intertwined. This is particularly the case with regards to social behaviours.

When animals with a mutation in a gene show a change in a specific behaviour, it is tempting to conclude that said gene is somehow involved in that behaviour. But this is not always the case. Animals are usually raised by their parents and grow up with siblings, who may share the same environment and genetic background (including this mutation). This makes it difficult to pinpoint exactly which elements, or combination of elements, are responsible for the emergence of these ‘behavioural phenotypes’ – that is, behaviours that are associated with a specific genotype.

To understand the direct effect of a specific mutation on the behavioural phenotype of an individual, the environment must be controlled for, including the genetic background of the individual’s social group – its genetic social environment (Baud et al., 2017). Now, in eLife, Rui Oliveira and co-workers based in Portugal, Israel and Poland – including Diogo Ribeiro as first author – report that, in zebrafish, the genetic social environment of an individual while it is growing up affects the adult’s behavioural phenotype (Ribeiro et al., 2020a).

Zebrafish are a good model to study the indirect effects of social genetic variation because they are highly social animals with a genome that can easily be modified. Ribeiro et al. first generated a mutant zebrafish line that lacks the gene for the oxytocin receptor, a protein involved in social-bonding behaviours in animals (Olff et al., 2013). A mutant fish was then either raised with its mutant siblings, or in a group of non-mutant fish. Similarly, a non-mutant individual was raised in a shoal of other non-mutants, or with mutant fish. Using different methods, the team then examined how each combination of genetic and social environment influenced the behavioural phenotype of the mutants.

Regardless of whether they were raised with mutants or non-mutants, fish that lacked the gene for the oxytocin receptor were always worse at discriminating between a familiar and an unfamiliar fish – a result predicted by previous studies (Ribeiro et al., 2020b). However, other experiments revealed that only mutant fish raised with other mutants were more reluctant to approach other fish and to integrate into a shoal. This showed that the genetic background of the group in which mutant fish were raised caused specific social phenotypes, as opposed to the loss of the oxytocin receptor gene alone.

This study may help researchers to understand how the genetic social environment can influence the impact of specific mutations on social interactions. It could also be relevant to work on other forms of behaviour, such as fear conditioning in mice: researchers wishing to investigate this behaviour would normally generate a mouse line lacking a gene thought to be involved in fear conditioning, and then examine how the mutation affects the behaviour of the mice. Variations in fear conditioning in the mutants would then be attributed to the genetic change rather than the social genetic environment. The work of Ribeiro et al. shows that researchers need to be aware of this effect, and control for it whenever possible.

These results also demonstrate the need to be cautious about the many human genetic studies that suggest potential links between a gene and the propensity to develop certain conditions. For instance, the general public now has easy access to DNA tests, which can link variations in certain genes to higher risks of becoming obese, being a smoker, or living a shorter life. However, a gene apparently associated with an increased risk for obesity may in fact be connected to increased parental anxiety. In this case, the weight gain would be a secondary effect of being raised by anxious parents. The impact of the social genetic environment should therefore be carefully assessed for all of these genes.

Finally, Ribeiro et al. show that specific social environments could potentially rescue or promote specific behavioural phenotypes, a finding that could be used to better study human behaviours and socialisation.
